# Children who develop allergy have low fecal alpha-defensin levels but high beta-defensin levels in infancy

**DOI:** 10.1186/2045-7022-1-S1-O32

**Published:** 2011-08-12

**Authors:** Emma Savilahti, Anna Kaarina Kukkonen, Tari Haahtela, Mikael Kuitunen, Erkki Savilahti

**Affiliations:** 1 University of Helsinki, Helsinki, Finland

## Background

Since early innate immunity responses and the intestinal flora guide adaptive immune responses, we investigated whether fecal defensin levels in infancy were associated with the emergence of allergy.

## Methods

In a randomized, double-blind placebo-controlled trial, 1018 infants in high risk for allergy received from birth to 6 months either a mixture of pre- and probiotics, or placebo. They were followed for the emergence of allergic diseases and sensitisation for 5 years. In an unselected group of 48 infants receiving probiotics and 52 receiving placebo, we measured fecal levels of human neutrophil peptide (HNP) 1-3, β-defensin 2 (HBD2) with enzyme linked immunosorbent assays (ELISA) at the age of 3 and 6 months. TNF-α and calprotectin had been measured with ELISA, and α1-antitrypsin with an immunodiffusion method in a proportion of samples.

## Results

Fecal levels of HNP1-3 and HBD2 decreased from 3 to 6 months. Low HNP1-3 and high HBD2 levels at 6 months were associated with allergy and sensitisation by the age of 5 years (p<0.05). HNP1-3 levels correlated negatively with α1-antitrypsin levels at the age of 3 months (coefficient -0.5; p<0.05) in children who developed sensitisation only or combined with allergic disorders. HBD2 levels correlated positively with TNF-α (0.7; p<0.05) in children with subsequent IgE-mediated allergy. Probiotic treatment tended (p<0.06) to increase fecal HBD2 levels at the age of 6 months compared with placebo.

## Conclusions

Early innate immunity responses in the gut are associated with the emergence of allergy later in childhood.

